# Human rabies in Israel.

**DOI:** 10.3201/eid0502.990227

**Published:** 1999

**Authors:** D David, C E Rupprecht, J Smith, I Samina, S Perl, Y Shram


ene (TCE), a major ground water contaminant used in the dry cleaning industry and in degreasing solvents.

The degree to which *B. cepacia* is being used in bioremediation products is unknown; however, the species has been used extensively to degrade ground water TCE contamination in at least one large U.S. city. A number of environment-friendly bioremediation products containing only naturally occurring, nonpathogenic bacteria are being marketed for use in drain opening and grease eradication systems. Because their formulations are proprietary, it is not known if these products contain *B. cepacia*; however, franchises that distribute such totally natural, noncorrosive, nontoxic products specifically target fast-food restaurants, photo processing facilities, and hospital radiology departments.

In the United States, the biopesticidal use of microorganisms such as *B. cepacia* is regulated by the Environmental Protection Agency (EPA) under the Federal Insecticide, Fungicide, and Rodenticide Act; however, the use of naturally occurring, nonpathogenic bacteria as bioremedial agents is essentially unregulated. Only new microorganisms (i.e., intergeneric or formed by combining genetic material from organisms in different genera) are regulated by EPA under the Toxic Substances Control Act (TSCA) (2). Ironically, TSCA regulations provide a strong disincentive to the development of safer microbiologic alternatives for use in bioremediation. For example, although the genetic elements responsible for TCE degradation by *B. cepacia* have been cloned, their recombination into another nonpathogenic bacterial host (e.g., *Escherichia coli*) would constitute a new microorganism, the licensure of which would be considered prohibitively time-consuming and expensive by many companies.

In Canada, biopesticidal uses of microorganisms are regulated by the Pest Management Regulatory Agency of Health Canada, under the Pest Control Products Act (PCPA); bioremedial uses are regulated by Environment Canada under the Canadian Environmental Protection Act (CEPA) (3). Both naturally occurring and genetically engineered microorganisms are strictly controlled under these acts. However, accurate species identification is the cornerstone of all notification of products under the Canadian regulations. This presents a further dilemma. At least five genomovars (discrete species) constitute what has recently been designated the "*B. cepacia* complex" (4). Insofar as the taxonomy of this group is poorly defined, there are no conventional taxonomic designations to distinguish pathogenic from nonpathogenic species. At present, it appears that all five *B. cepacia* genomovars are capable of causing infections in vulnerable persons (4).

Because the epidemiology of *B. cepacia* complex infection in humans is incompletely understood, the threat posed by the inclusion of this species in biopesticides and bioremedial products is difficult to quantify. However, we agree with Holmes et al. that such use should be approached with considerable caution. In a broader context, the commercial use of *B. cepacia* illustrates our incomplete understanding of nonpathogenic bacteria and their potential to cause human disease. Regulations governing the use of microorganisms in industry must constantly adapt to keep pace with the emergence of infections due to nonpathogens and limit risk to human health.

**John J. LiPuma* and**
**Eshwar Mahenthiralingam†**
*MCP Hahnemann University, St Christopher's Hospital for Children, Philadelphia, Pennsylvania, USA; and †University of British Columbia, Vancouver, British Columbia, Canada

### References

1. Holmes A, Govan J, Goldstein R. Agricultural use of *Burkholderia (Pseudomonas) cepacia*: a threat to human health? Emerg Infect Dis 1998;4:221-7.
2. Microbial products of biotechnology; final regulations under the Toxic Substances Control Act; final rule. Washington: U.S. Environmental Protection Agency, 1997. Federal Register. Vol 62. p. 17910-58.
3. Guidelines for the notification and testing of new substances: organisms. Pursuant to the New Substances Notification Regulations of the Canadian Environmental Protection Act. Ottawa, Canada: Environment Canada, Health Canada, 1997.
4. Vandamme P, Holmes B, Vancanneyt M, Coenye T, Hoste B, Coopman R, et al. Occurrence of multiple genomovars of *Burkholderia cepacia* in cystic fibrosis patients and proposal of *Burkholderia multivorans* sp. nov. Int J Syst Bacteriol 1997;47:1188-200.

### Human Rabies in Israel

**To the Editor:** Rabies, a major zoonotic disease in the Middle East, has two main epidemiologic forms: urban and sylvatic. The last case of human rabies in Israel was in the Golan Heights in 1971 (1). Twenty-five years later, in 1996, rabies was reported in a 20-year-old soldier, and then two cases were documented in 1997.

The first case-patient, a soldier in the Golan Heights, was bitten on the lip by an unidentified animal while sleeping. The wound was cleansed and sutured; clinical signs started 39 days later with high fever and headache. The patient was admitted to an emergency room with hallucinations, difficulty in swallowing, and generalized weakness, and rabies was considered in the differential diagnosis; 3 days later the patient became comatose. Samples of saliva, serum, and cerebrospinal fluid; skin biopsy tissue; and corneal impressions were sent to the Pasteur Institute, Paris, France. Eight days after clinical signs developed, rabies was diagnosed by the Kimron Institute by heminested reverse transcription-polymerase chain reaction (hnRT-PCR) on the patient's saliva (2). The RT step was performed with the specific primer 113 (5'-GTAGGATGATATATGGG-'3 at 1013-1030), followed by PCR with the 509 (5'-GAGAAAGA ACTTCAAGA-'3 at 1156-1173) and 304 (5'-GAGT CACTCGAATATGTC-'3 at 1513-1533) primers. The hn-PCR was performed with the 509 and 105 (5'-TTCTTATGAGTCACTCGAATA TGTCTTGTTTAG-'3 at 1393-1426) primers (3). The PCR results were confirmed by the Pasteur Institute 3 days later, and the patient died 35 days after clinical symptoms appeared.

The second case-patient was a 7-year-old girl admitted to the hospital unconscious. Two months before admission, she had been scratched while sleeping by an unidentified animal. On the second hospital day, generalized convulsions and gasping occurred. During the following days, brain stem function progressively deteriorated. Rabies was diagnosed by hnRT-PCR on the saliva sample, and the diagnosis was confirmed by the Centers for Disease Control and Prevention (CDC). The patient died despite supportive care.

The third case-patient, a 58-year-old man with fever, headache, and sore throat, was diagnosed as having pharyngitis and received an oral antibiotic. The patient had been bitten 3 months earlier while sleeping. On admission, the lumbar puncture, computerized tomography scan, and electroencephalogram were normal. On the third hospital day, he had respiratory arrest; during orotracheal intubation, acute laryngospasm with copious amounts of salivation occurred. Rabies was suspected, and viral RNA in the saliva was detected by hnRT-PCR. One day later the patient died.

We injected antemortem saliva and postmortem brain tissue from these patients into suckling mice intracerebrally. Virus was isolated from saliva samples of case-patients 1 and 3 but not from the sample of case-patient 2. Rabies virus antigen in the brain tissue was confirmed by direct immunofluorescence assay, and viral RNA was detected by RT-PCR.

For genetic analysis, we used brain samples from the three case-patients and from animals that died of rabies near the location of the case-patients to amplify and sequence a 328-bp (264 bp from the 3' of the N gene and 64 bp of the 3' NS-N region) fragment. On the basis of homologic results of nucleotide sequences in the three case-patients and in virus isolates from animals in the same regions, we concluded that a reservoir for rabies in foxes is responsible for infection of all three humans.

The three human isolates were tested with a panel of 19 anti-N protein monoclonal antibodies (CDC, Atlanta, GA, USA) and compared with those of rabies isolates from the geographic vicinity of the human cases. Isolates from case-patients 1 and 3 belonged to variant 1 (MAb C18 negative) and were similar to virus isolates from 10 foxes, one jackal, and four cattle in the same regions. Isolates from case-patient 2 belonged to antigenic variant 2 (MAbs C2, C7, C12, C13, C18 negative) and were similar to isolates from four foxes, one dog, and one cow in the vicinity of the second case-patient.

Early antemortem diagnosis of virus in an infected human is very important. Checking for virus in saliva eliminates the difficulty of tissue sampling from humans with suspected cases of rabies, and the sensitivity of hnRT-PCR makes it the technique of choice for detecting limited amounts of virus. Previous work showed that a 200-bp region of the N gene had only one nucleotide difference between them (4). Moreover, two samples from a region in western Mexico, isolated 30 years apart, were identical in sequence (4). Incorporation of the reference strains Pasteur and SAD B19 into our phylogenetic tree indicated that the three human viruses we isolated belong to lyssavirus genotype 1.

**Dan David,* Charles E. Rupprecht,† Jean Smith,† Itzhak Samina,* Shmuel Perl,* and Yehuda Stram***
*Kimron Veterinary Institute, Bet Dagan, Israel; and †Centers for Disease Control and Prevention, Atlanta, Georgia, USA

### References

1. Shimshony A. Veterinary public health in Israel. Revue Scientifique et Technique Office International Des Epizooties 1992;11:77-98.
2. Heaton PR, Johnstone P, McElhinnely M, Coweley R, O'Sullivan E, Whitby JE. Heminested PCR assay for detection of six genotypes of rabies and rabies related viruses. J Clin Microb 1997;35:2762-6.
3. Smith JS. Rabies virus. In: Murray PR, Baron EJ, Pfaller MA, Tenover FC, Yolken RH, editors. Manual of clinical microbiology. 6th ed. Washington: American Society for Microbiology; 1995. p. 907-1003.
4. Smith JS, Seidel HD, Warner CK. Epidemiology and historical relationships among 87 rabies virus isolates determined by limited sequence analysis. J Infec Dis 1992;166:296-307.

### Emerging Infections and Disease Emergence

**To the Editor:** Emerging infections have been defined as diseases whose incidence in humans has increased within the last 2 decades or threatens to increase in the near future (l). This definition, with minor variations, has continued to be used, although occasional debate erupts over whether one disease or another is truly emerging. Use of the term "emerging" has facilitated communication about the changed pattern of infectious diseases in recent years. While the study of infectious organisms and clinical training about emerging infections are manifestly necessary, they are not a sufficient foundation for understanding the process of disease emergence. I would propose, specifically, that we distinguish between emerging diseases—the study of specific infections that are changing—and the study of disease emergence.

Studies of emerging infections typically rely on disease, organismic, or syndromic approaches. Meetings on emerging infections typically cover newly recognized or characterized organisms or diseases and update information about the recognition, diagnosis, treatment, prevention, and control of these infections. This ongoing education is essential for practicing clinicians, who finished their formal training before AIDS, Lyme disease, ehrlichiosis, *Helicobacter pylori* infection, cryptosporidiosis, cyclosporiasis, and many other infections were described. These meetings also help clinicians learn how to fit new information into their existing knowledge base: What is the probability that a person with rash and fever has ehrlichiosis and that a person with fever and pulmonary infiltrates has hantavirus pulmonary syndrome?

By contrast, understanding the process of disease emergence involves studying the origins and ecology of emerging infections. Many disciplines relevant to disease emergence lie outside traditional infectious disease training and research and include evolutionary biology, demography, population dynamics, ecology, vector biology, climatology, epidemiology, genetics, veterinary medicine, and behavioral sciences (2). Infectious diseases of animals and plants have both a direct and indirect impact on human health. The study of infectious diseases in other species may provide important insights into understanding the process of disease emergence in humans. The study is also relevant to understanding the species-to-species spread of organisms.

Tools used to study and understand disease emergence include mathematical modeling, geographic information systems, remote sensing, molecular methods to study the genetic relatedness of organisms, and molecular phylogeny. Paleobiology, paleoecology, and studies that allow the reconstruction of past events may help inform future research and policy.

A major challenge is to reach people with relevant skills, knowledge, and experience and develop a coherent framework to advance the understanding of the process of disease emergence. No one institution, organization, or country can itself prevent or manage emerging infectious diseases.

In the study of emerging infections we focus on the organism, the patient, and the human population. The study of disease emergence must be at the systems level and must look at ecosystems, evolutionary biology, and populations of parasites and hosts, whatever their species. A primary goal should be to identify conditions or combinations or sequences of events that herald a changed pattern of infections so that preventive strategies can be used.

